# Role of CORO1A in Regulating Immune Homeostasis of Mammary Glands and Its Contribution to Clinical Mastitis Development in Dairy Cows

**DOI:** 10.3390/biom15060827

**Published:** 2025-06-06

**Authors:** Bohao Zhang, Na Chen, Xing Yu, Jianfu Li, Weitao Dong, Yong Zhang, Xingxu Zhao, Quanwei Zhang

**Affiliations:** 1 College of Veterinary Medicine, Gansu Agriculture University, Lanzhou 730070, China; zhangbhgs@163.com (B.Z.); dongwt@gsau.edu.cn (W.D.); zhangyong@gsau.edu.cn (Y.Z.); zhaoxx@gsau.edu.cn (X.Z.); 2 College of Life Science and Technology, Gansu Agriculture University, Lanzhou 730070, China; 1073323020311@st.gsau.edu.cn (N.C.); 1073324020326@st.gsau.edu.cn (X.Y.); lijfu@st.gsau.edu.cn (J.L.); 3 Gansu Key Laboratory of Animal Generational Physiology and Reproductive Regulation, Lanzhou 730070, China

**Keywords:** immune homeostasis, clinical mastitis, coronin1A, bioinformatics analysis

## Abstract

Immune homeostasis refers to the immune system’s ability to maintain a dynamic balance, defend against infections while preventing excessive inflammation, and preserve normal physiological activity. However, its regulatory role in the mammary glands (MGs) of cows with clinical mastitis (CM) remains unclear. This study examined MG tissue samples collected from healthy Holstein cows and those with CM caused by *Staphylococcus aureus* (*n* = three per group) to identify candidate biomolecular targets involved in immune homeostasis in dairy cows affected by mastitis through a proteomics-based bioinformatic analysis and analyze their expression and localization in MG tissues. A pathological examination revealed that the MG tissues of the CM group exhibited significant alveoli collapse and inflammatory cell infiltration. The presence of activated phagolysosomes and lysosomes indicated active immune and phagocytic responses. Bioinformatics highlighted coronin1A (CORO1A) as a potential modulator of immune responses through phagosome formation. Dysregulation could impair immune homeostasis, thereby exacerbating mastitis. Immunofluorescence and immunohistochemistry staining showed that CORO1A was localized in monocytes, macrophages, and neutrophils. Molecular mechanism analysis revealed that Toll-like receptor 2 (TLR2) recognizes pathogens and recruits CORO1A to the phagosome formation site, thereby enhancing the phagocytic activity of immune cells. The expression levels of *CORO1A* and *TLR2* mRNA and proteins were positively correlated with the incidence of mastitis. In conclusion, CORO1A upregulation may activate immune and phagocytic responses, disrupting MGs’ immune homeostasis during *Staphylococcus aureus*-induced mastitis. These findings provide novel insights into mastitis pathogenesis and potential therapeutic targets.

## 1. Introduction

Clinical mastitis (CM) in dairy cows is characterized by the acute inflammation of the mammary glands (MGs), typically presenting with clear symptoms, including redness, fever, and abnormal milk production [1]. CM compromises mammary health, reduces milk yield, and significantly influences the global dairy industry, incurring substantial economic losses owing to increased artificial breeding costs, medical treatments, and dairy waste [2,3]. Recent research has led to significant progress in understanding the pathogenesis of CM, including the identification of key bacterial pathogens, immune responses, and genetic factors associated with susceptibility [4]. *Staphylococcus aureus*, a Gram-positive bacterium, is a common cause of recurrent mastitis, with virulence factors like protein A and alpha toxin contributing to mammary tissue damage and increased antibiotic resistance complicating treatment [5]. *Escherichia coli*, a Gram-negative bacterium, often leads to acute mastitis with a sudden onset and severe symptoms [3,6]. The mechanisms by which these bacteria induce mastitis involve complex interactions with mammary tissues, which have been increasingly understood through multi-omics approaches, revealing activation patterns of key inflammation-related pathways and providing deeper insights into host–pathogen interactions [1,7]. However, notwithstanding recent advancements, CM remains a costly and persistent challenge in dairy production. Therefore, exploring the pathological and molecular mechanisms underlying the formulation of novel approaches to prevent and treat CM is essential.

Immune homeostasis in MG tissues is crucial for regulating inflammatory cytokine secretion and immune cell activation, which prevents pathogen invasion, while controlling the spread of inflammation [6,8]. In fact, the dysregulation of immune homeostasis is a significant contributor to mammary tissue damage. For example, pattern recognition receptors (PRRs) detect invading pathogens in mammary epithelial cells (MECs) and initiate innate immune responses [7,8]. However, excessive immune activation disrupts this balance, triggering the overproduction of inflammatory mediators and exacerbating inflammation and tissue damage [9]. Furthermore, dysregulated immune homeostasis may promote autophagy and apoptosis, further exacerbating the tissue pathology. Studies show that the inflammatory response triggered by pathogen infection during CM disrupts homeostasis by altering leukocyte numbers and activity, leading to the infiltration of mammary tissue [10]. For instance, neutrophils rapidly recognize and engulf pathogens; however, the release of reactive oxygen species (ROS) may cause collateral damage to MECs [11]. During pathogen infection and the progression of mastitis, monocytes are recruited to the mammary gland tissues early in the inflammatory response. Upon arrival, these monocytes differentiate into macrophages and dendritic cells, playing a crucial role in pathogen clearance, antigen presentation, and cytokine secretion [5]. Macrophages, through the phagocytosis of pathogens and dead cells, contribute to local immune modulation by both clearing pathogens and regulating the inflammatory response. However, dysregulated macrophages may shift from an inflammatory state to a more immune-suppressive phenotype, potentially impairing effective tissue repair [5]. This monocyte recruitment and differentiation process is most pronounced during the early to acute stages of infection or mastitis, contributing to both inflammation and tissue damage [5]. Moreover, the immune homeostasis of MECs can be directly disrupted by abnormalities in ion balance and transport, thereby impairing the health and function of mammary tissue [12]. Under normal conditions, cells rely on channels and transporters to regulate balance, which is essential for cellular functions, signal transduction, and inflammatory responses [13]. However, in CM, compromised MEC barrier function leads to imbalances in calcium (Ca^2+^), sodium (Na^+^), and hydrogen ions (H^+^), thereby disrupting cellular metabolism and immune responses [14,15]. For instance, inflammatory responses often cause H^+^ imbalance and local tissue acidification. Changes in intracellular pH can disrupt lysosomal enzyme activity, impairing the ability of the body to clear pathogens and affecting tissue immunity and repair mechanisms [10]. In conclusion, the regulation of immune homeostasis is crucial to the pathophysiological development of mastitis in bovines. Thus, investigating the molecular mechanisms underlying immune homeostasis regulation is essential for comprehending normal MGs’ development and developing novel therapeutic strategies for mastitis.

The disruption of mammary immune homeostasis in dairy cows is initiated by pathogen invasion, in which pathogen-associated molecular patterns (PAMPs) are sensed by PRRs on MECs and immune cells [7,16]. Specifically, the activation of Toll-like receptors (TLRs) and NOD-like receptors (NLRs) triggers downstream signaling cascades [17]. TLR4-triggered signaling through the MyD88 adaptor protein initiates the phosphorylation of TNF receptor-interacting factor 6 (TRAF6), thereby promoting the NF-κB and MAPK pathways’ activation, which facilitates the rapid release of pro-inflammatory factors such as interleukin (IL)-1β and IL-6 [17,18]. Simultaneously, activating the NLRs’ inflammasome promotes the maturation and release of IL-1β and IL-18, further amplifying the inflammatory response and rapidly disrupting local immune homeostasis [17]. The sustained overexpression of these pro-inflammatory cytokines further strengthens NF-κB and Janus kinase/signal transducer (JAK) pathways, creating a self-reinforcing cycle that exacerbates inflammation, while suppressing anti-inflammatory mediators [19,20]. This imbalance skews the T helper 17 (Th17)/regulatory T (Treg) cell axis toward a pro-inflammatory phenotype, intensifying immune dysregulation [21]. Additionally, excessive neutrophil infiltration into the mammary tissue, driven by C-X-C motif ligand 8 (CXCL8)-mediated chemotaxis, leads to the release of myeloperoxidase, elastase, and ROS, which, although contributing to pathogen clearance, also cause collateral damage to MECs [16]. Furthermore, macrophage polarization shifts towards the pro-inflammatory M1 phenotype, thereby exacerbating tissue inflammation and impairing the repair function of M2 macrophages [22]. Ultimately, this immune dysregulation results in chronic inflammation and persistent cellular damage, compromising MGs’ function and reducing milk production. Given these detrimental effects, the systematic identification of biomolecular targets involved in immune homeostasis regulation and an advanced comprehension of their underlying mechanisms are critical for creating targeted therapies for bovine mastitis.

This research was designed to comprehensively determine Gene Ontology (GO) terms and Kyoto Encyclopedia of Genes and Genomes (KEGG) pathways, candidate differentially expressed proteins (DEPs), and target molecules related to immune homeostasis in Holstein cows affected by CM through a bioinformatics analysis of data-independent acquisition (DIA) proteomics data. Additionally, changes in the expression and localization of immune homeostasis-associated genes and proteins were evaluated in MG tissues from healthy and CM-affected cows. Our findings offer a novel understanding of the mechanisms behind immune homeostatic regulation and target molecules in the case of mastitis.

## 2. Materials and Methods

### 2.1. Sample Preparation and Collection

**Figure 1:**
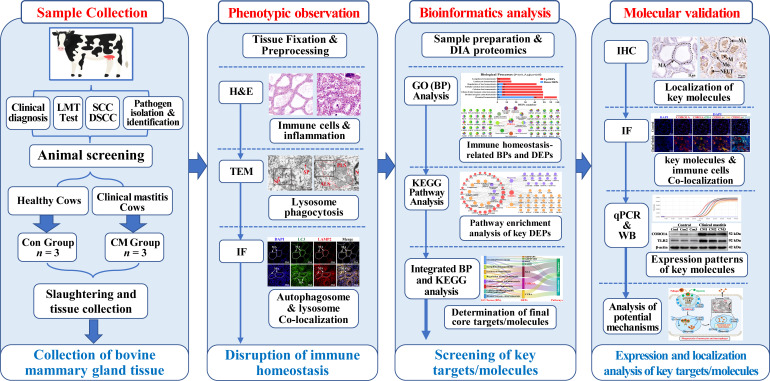
Experimental design and methodology flowchart.

Holstein cows (5–6 years old, 3rd to 5th lactation, 60–250 days in milk) with matched parity, uniform milking systems, and comparable body condition scores were selected from a privately operated dairy facility (Wuzhong City, Ningxia Hui Autonomous Region, China). These cows were housed in freestall barns under identical management conditions with ad libitum water access and underwent MG evaluations based on standard veterinary clinical diagnostic criteria for abnormal milk and signs of inflammation, including redness, elevated temperature, swelling, and discomfort [23]. Milk samples (45–50 mL) were collected under sterile preservative-treated conditions in centrifuge tubes for the somatic cell count (SCC) analysis, differential somatic cell count (DSCC), Lanzhou Mastitis Test (LMT), and pathogen isolation and identification (Supplementary Figure S1) [24]. Holstein cows in the control (Con) group without pathogens and cows with CM induced using *Staphylococcus aureus* were screened following established protocols [23]. After screening, the cows that met the inclusion criteria were transported to a slaughterhouse where sterile surgical scissors were used to excise the connective and adipose tissues post-slaughter. Fresh MG tissues were collected from the two groups (*n* = 3/group). Tissue samples were prepared by sectioning them into 1 cm^3^ pieces for fixation in 4% paraformaldehyde and 1 mm^3^ pieces for fixation in 2.5% glutaraldehyde. The remaining tissue was frozen in liquid nitrogen for storage at −80 °C. This research received ethical approval from the Ethics Committee of Gansu Agriculture University, Lanzhou, China (No. GSAU-Eth-VMC-2021-020 and approval date 11 March 2021). A schematic representation of the experimental design is provided in Figure 1.

### 2.2. Hematoxylin–Eosin (H&E) Staining

MG tissues were fixed, paraffin-embedded, and sectioned using a microtome (Leica, Wetzlar, Germany). The sections were baked, deparaffinized, and dehydrated. H&E staining was subsequently performed following established protocols [25]. After sealing them with neutral balsam (Solarbio, Beijing, China), images were acquired using an OLYMPUS microscope and imaging system (Tokyo, Japan). All assays were conducted at least three times.

### 2.3. Transmission Electron Microscopy (TEM)

TEM was employed to assess morphological alterations in cell vesicles and lysosomes within the MGs. As described previously [26], following fixation, rinsing, and dehydration at 25 °C, the tissues were infiltrated and embedded in resin blocks for ultrathin sectioning (60–80 nm thick). Sections were mounted onto 150-mesh copper grids coated with Formvar film and stained with 2% and 2.6% uranyl acetate and lead citrate, respectively. After drying them with filter paper, the grids were placed on a grid holder and left to dry overnight. Electron micrographs were obtained with a Hitachi HT7800 TEM (Hitachi, Minato-Ku, Japan).

### 2.4. Immunofluorescence (IF) Staining

IF staining procedures were carried out as previously described [23,27]. Tissue sections were incubated with primary antibodies, including rabbit anti-Microtubule-associated proteins 1A/1B light chain 3 (LC3), anti-Lysosome-associated membrane glycoprotein 2 (LAMP2), anti-CD14, anti-CD68, anti-CD11β (Servicebio, Wuhan, China), and anti-coronin1A (CORO1A, Proteintech, Wuhan, China), at varying concentrations (Supplementary Table S1). Nuclei were counterstained with 4′,6-diamidino-2-phenylindole (DAPI; Solarbio, Beijing, China), and images were captured using fluorescence microscopy (OLYMPUS, Tokyo, Japan). For each tissue sample, at least three non-overlapping fields were selected under identical exposure settings. Integrated optical density (IOD) was calculated as the product of the stained area and mean gray value for each region of interest, following background subtraction. The values from all fields per sample were averaged for statistical comparison. The IOD of positive expression products was measured using ImageJ software v1.44p (NIH; Bethesda, MD, USA). All IF assays were conducted at least three times to ensure reproducibility.

### 2.5. Bioinformatics Analysis

DIA proteomic data deposited in the Proteome Exchange database (accession numbers IPX0003382000/PXD028100) were subjected to GO terms and KEGG pathway annotation analyses. GO annotation analysis was conducted using the official Gene Ontology database (http://geneontology.org, accessed on 25 February 2024), and KEGG analysis was based on the KEGG database (https://www.genome.jp/kegg/, accessed on 25 February 2024). This study primarily focused on DEPs classified under GO terms (*p* < 0.05) associated with biological processes (BPs) related to immune homeostasis. After identifying overlapping DEPs, KEGG enrichment analysis (*p* < 0.05, *Q* < 0.05) was performed to explore their functional roles. Heatmaps, Volcano plots, Venn diagrams, radar charts, circular graphs, and Sankey diagrams were generated using R programming language (version 4.3.3) and online OmicShare tools (https://www.omicshare.com/tools/, accessed on 20 March 2024) [23,27]. Additionally, a protein–protein interaction (PPI) network of key DEPs was generated through STRING v.12.0 (EMBL, Heidelberg, Germany) and Cytoscape 3.9.1, including the ClueGO plugin (Cytoscape Consortium, La Jolla, CA, USA) [23,28].

### 2.6. Immunohistochemistry (IHC) Staining

Following sequential deparaffinization and rehydration, sections were soaked and heated in citrate buffer (Solarbio, Beijing, China) for antigen retrieval. Tissue sections were then treated with endogenous peroxidase blocking buffer (Beyotime, Shanghai, China) and blocked in blocking solution at 25 °C. The sections were incubated at 4 °C with rabbit anti-CORO1A (Proteintech, Wuhan, China). After primary antibody incubation, IHC staining was performed in accordance with the manufacturer’s protocol [28,29]. Images were acquired using a microscope (Nikon, Tokyo, Japan), and the IOD of positive signals was quantified with ImageJ software (NIH, Bethesda, MD, USA). All assays were conducted at least three times.

### 2.7. RNA Isolation, cDNA Synthesis, and Quantitative Polymerase Chain Reaction (qPCR) Assays

Total RNA was extracted from the tissues using TransZol Up (TransGen, Beijing, China), which was subsequently used for cDNA synthesis. Total RNA (1 µg) was reverse-transcribed into single-stranded cDNA using the Evo M-MLVRT kit for qPCR II (AG; Changsha, China), following the instructions of the manufacturer [23,28]. The relative expression levels of *CORO1A* and *TLR2* mRNA in tissues were quantified using qPCR, with *β-actin* serving as an endogenous control. Primers for qPCR assays (Supplementary Table S2) were generated using Primer3 (v4.1.0) and synthesized by Qingke Biotech (Xi’an, China). The qPCR was conducted on a LightCycler system, and the data were analyzed using the 2^−ΔΔCT^ method, as previously described [30]. All qPCR reactions were performed in triplicate.

### 2.8. Western Blot (WB) Analysis

The relative expression of CORO1A and TLR2 proteins in MGs of the C/CM groups was assessed by Western blotting. Proteins were isolated from 80 mg tissue samples using radioimmunoprecipitation assay buffer (TransGen, Beijing, China), and concentrations were measured using a bicinchoninic acid assay kit (Bioss, Beijing, China). WB analysis was conducted according to the method previously described [27,28]. During electrophoresis, Blue Plus II Protein Marker (TransGen, Beijing, China) was used as a molecular weight reference (Supplementary Figure S2). Rabbit anti-CORO1A, mouse anti-TLR2 (Proteintech, Beijing, China), and β-actin (Bioss, Beijing, China) were incubated with the samples overnight at 4 °C. Band intensity was quantified using ImageJ (NIH, Bethesda, MD, USA), with β-actin used as a normalization control. All WB analyses were performed in triplicate. Western blot original images can be found in Supplementary Materials.

### 2.9. Statistical Analysis

All results are expressed as mean ± standard deviation, unless specified otherwise. Statistical analyses were conducted using SPSS v22.0 (SPSS Inc., Chicago, IL, USA). For qPCR and WB data, Student’s *t*-test was applied to compare two groups, while one-way ANOVA was used for comparisons among multiple groups. Graphs were generated using GraphPad Prism 9.0 (San Diego, CA, USA) and Adobe Illustrator 2022 software (Adobe Inc., San Jose, CA, USA). *p* value of less than 0.05 was considered statistically significant.

## 3. Results

### 3.1. Histologic and Ultrastructural Pathology Observation of the MGs in Holstein Cows with CM

**Figure 2:**
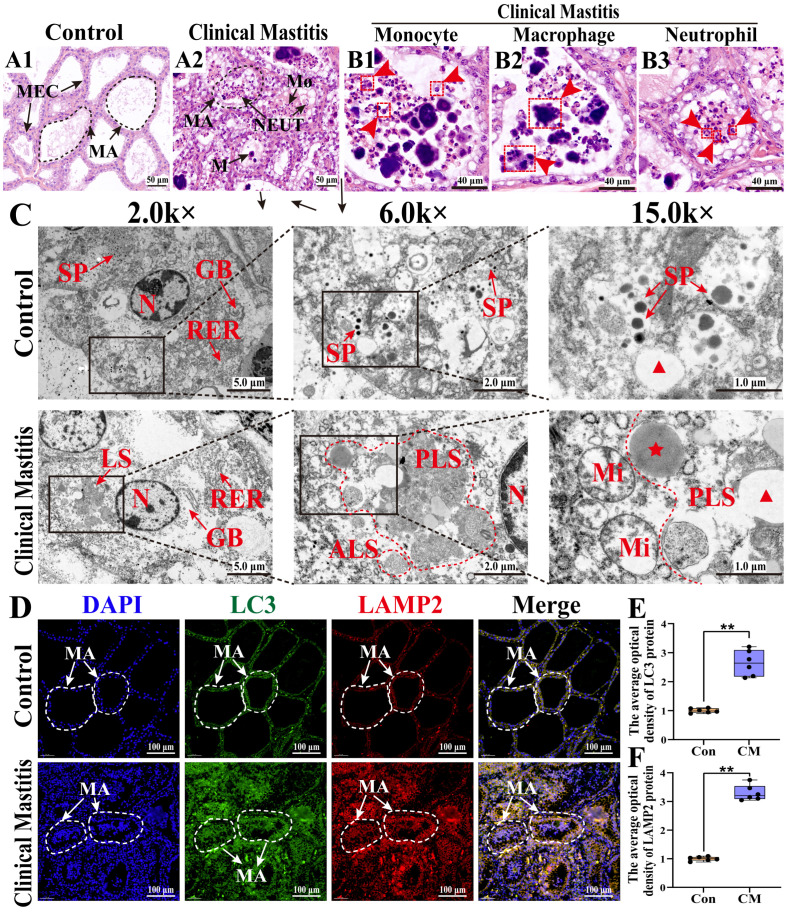
Phenotypes of pathological phenomena in the dairy cows’ MGs of the C and CM groups (*n* = 3/group). (**A1**,**A2**) Morphological structures of the alveoli in MGs were observed using H&E staining. (**B1**–**B3**) Different types of immune cells in MGs of the CM group. (**C**) Ultrastructural changes in MGs observed using TEM. (**D**) IF staining of LC3 and LAMP2 proteins: nuclei (blue), LC3 (green), LAMP2 (red), and merged LC3 and LAMP2 staining. (**E**,**F**) The IOD of LC3 (**E**) and LAMP2 (**F**) signals quantified using ImageJ software. MA, mammary alveoli; MEC, mammary epithelial cell; NEUT, neutrophil; M, monocyte; MΦ, macrophage; N, nucleus; GB, Golgi body; LS, lysosome; PLS, phagolysosome; ALS, autophagic lysosome; RER, rough endoplasmic reticulum; SP, secretory granule. Red pentagram represents the primary lysosome. Red triangle represents the cellular vesicle. Scale bars: 100, 50, 40, 5, 2, and 1 μm, represent 100×, 200×, 800×, 2.0k×, 6.0k×, and 15.0k, respectively. All images in this figure are representative. ** represents *p* < 0.01.

To investigate immune homeostasis during inflammatory stress, the histopathological changes in MG tissues were examined in both groups (Figure 2). The H&E staining showed that those alveolar structures remained intact, with well-preserved mammary alveoli (MA) and neatly arranged MECs in the Con group (Figure 2(A1)). In contrast, the alveoli were collapsed, coupled with infiltrated inflammatory cells in the MGs of the CM group (Figure 2(A2)). Consistently, an extensive presence of monocytes, macrophages, and neutrophils was observed in the CM group (Figure 2(B1–B3)). The TEM analysis demonstrated distinct ultrastructural differences between the two groups (Figure 2C). Specifically, the well-organized rough endoplasmic reticulum (RER) and Golgi bodies facilitated protein synthesis, whereas the presence of secretory granules confirmed the active secretory function of MG tissues in the Con group. In contrast, the disrupted homeostasis in the CM group was characterized by enhanced lysosomal phagocytic activity, as evidenced by the presence of phagolysosomes and autophagic lysosomes, along with fragmented RER, vacuolated mitochondria, and primary lysosome formation, indicating immune phagocytic activation. The IF results revealed that LC3 (an autophagosome marker) and LAMP2 (a lysosome marker) were expressed and co-localized in MECs and immune cells within the MG tissues of both groups (Figure 2D), with substantially increased expression in the CM group (*p* < 0.01, Figure 2E,F). These findings indicate that CM disrupted the immune homeostasis in MG tissues by inducing immune phagocytosis, characterized by autophagosome formation, lysosomal activation, and immune cell infiltration.

### 3.2. Screening Immune Homeostasis-Associated GO Terms and DEPs

**Figure 3:**
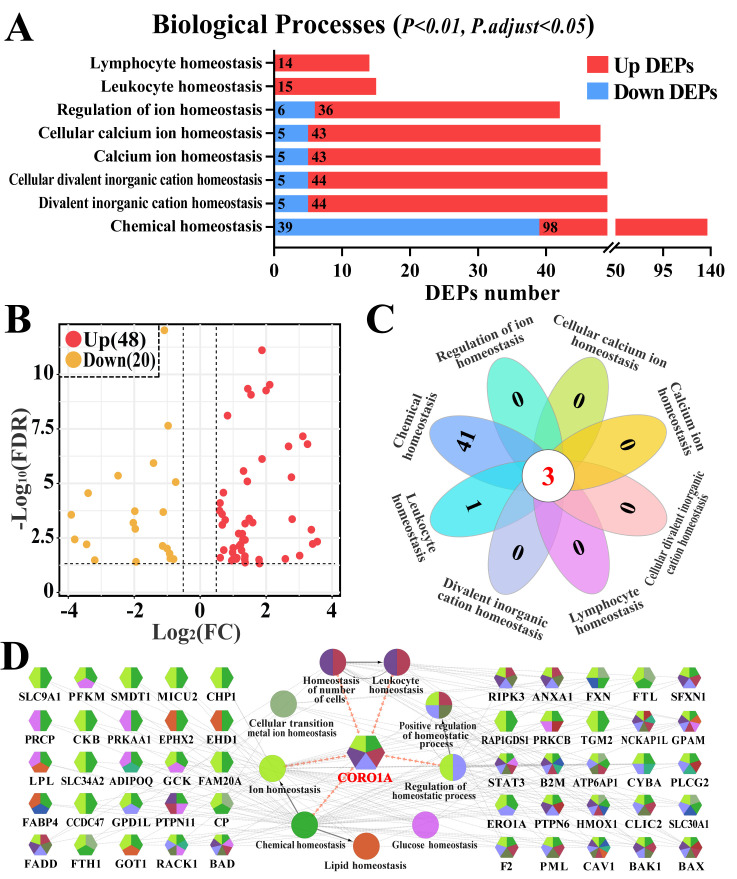
Candidate DEPs and enriched Gene Ontology (GO) terms associated with immune homeostasis. (**A**) Candidate differentially expressed protein (DEP) and BP term analysis associated with immune homeostasis. x-axis represents the number of DEPs. y-axis represents the BP terms. (**B**) Volcano plot displaying 68 DEPs, including 20 down- and 48 upregulated DEPs associated with eight BP terms. (**C**) Venn diagram illustrating the eight BP terms. (**D**) PPI network analysis depicting the relationship between BP terms and the candidate DEPs using ClueGO.

Homeostasis, especially immune homeostasis-related GO terms, and candidate DEPs were screened and further analyzed (Figure 3, Supplementary Table S3). Eight BP terms (*p* < 0.01 and *p. adjust <* 0.05), including immune cell-related homeostasis (such as leukocyte and lymphocyte homeostasis) and ion-related homeostasis (such as calcium ion and divalent inorganic cation homeostasis) were identified (Figure 3A). After accounting for the overlapping DEPs, 68 DEPs were identified among the eight BP terms (Supplementary Table S4). The volcano plot highlighted that 20 of the 68 identified DEPs were downregulated, whereas 48 were upregulated (Figure 3B). The Venn diagram further indicated that three DEPs (BAX, BAK1, and CORO1A) were shared among the eight BPs (Figure 3C). The PPI network revealed that 50 of the 68 DEPs were directly involved in nine BPs, and CORO1A, a pivotal candidate DEP, participated in five BPs related to leukocyte and ion homeostasis (Figure 3D). These findings indicate that the identified DEPs influenced CM progression by regulating immune homeostasis and that CORO1A may play pivotal roles in this process.

### 3.3. Screening CORO1A-Associated KEGG Pathways and DEPs

**Figure 4:**
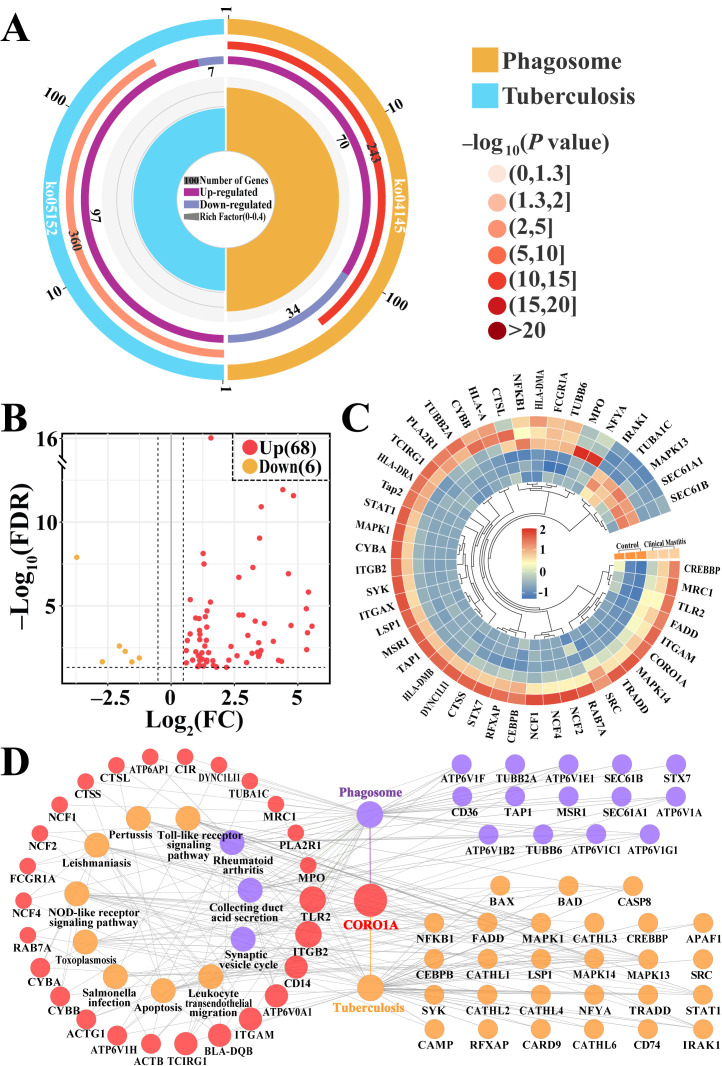
KEGG pathway analysis of CORO1A-related DEPs and pathways. (**A**) Enrichment circle diagram of the significantly different pathways associated with CORO1A. (**B**) Volcano plot displaying 74 DEPs including six down- and 68 upregulated DEPs associated with two pathways. (**C**) Heatmap of the 74 DEPs. (**D**) PPI network of the DEPs across the two pathways using ClueGO.

CORO1A-related pathways and key DEPs were screened and further analyzed (Figure 4, Supplementary Table S5). The enrichment circle diagram shows that two pathways related to CORO1A, phagosome and tuberculosis, required further investigation (Figure 4A). The Volcano plot and heatmap analysis revealed that these 74 key DEPs, including six downregulated and 68 upregulated, were significantly differentially expressed in these two groups (Figure 4B,C). The PPI network demonstrated that 68 of the 74 DEPs were closely associated with 13 KEGG pathways, highlighting its involvement in immune response, apoptosis, and various disease pathways. Notably, CORO1A was linked to phagosome formation, tuberculosis, and immune-related pathways such as the TLR pathway and nucleotide-binding oligomerization domain NLR signaling pathway (Figure 4D). These findings indicate that the identified pathways and DEPs, particularly CORO1A, played a crucial role in immune regulation and phagocytosis during MG tissue inflammation in Holstein cows.

### 3.4. Conjoint Analysis of Candidate BP Terms and Pathways Associated with CORO1A

**Figure 5:**
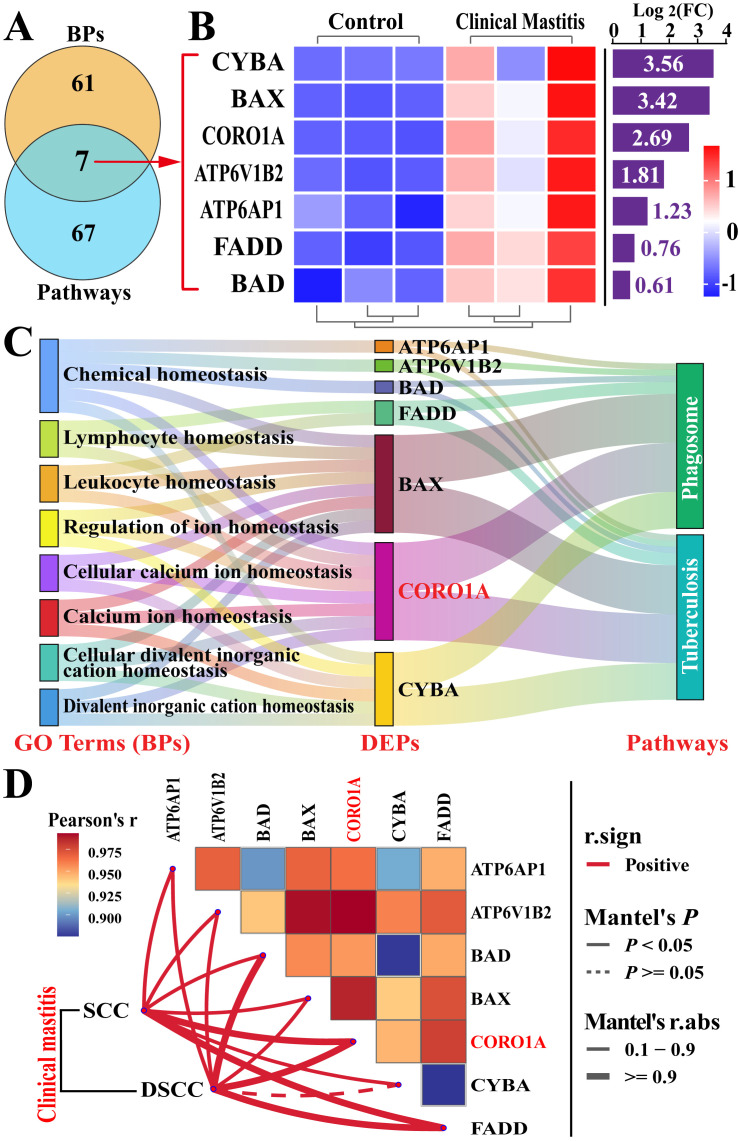
Candidate DEPs associated with CORO1A identified through enrichment analyses of BPs and pathways. (**A**) Venn diagram illustrating DEPs within BP terms and pathways associated with CORO1A. (**B**) Heatmap and relative expression levels of seven quantified DEPs. (**C**) Sankey diagram mapping the eight BPs, seven shared DEPs, and two pathways. (**D**) Correlation analysis between candidate DEPs in MG tissues and SCC/DSCC in milk samples. SCC, somatic cell count; DSCC, differential somatic cell count.

Significant candidate DEPs interacting with CORO1A were identified through the analysis of BPs and pathways (Figure 5, Supplementary Table S6). A Venn diagram showed that seven candidate DEPs were co-identified across eight BPs and two pathways (Figure 5A). These DEPs exhibited differential expression between the two groups, with pronounced upregulation in the CM group (Figure 5B). The functional relationships between key DEPs, BP terms, and pathways associated with immune homeostasis regulation were further analyzed using a Sankey diagram, which revealed the broad involvement of DEPs across multiple pathways and BPs, with CORO1A playing a particularly prominent role (Figure 5C). A correlation analysis between seven DEPs, SCC, and DSCC revealed that six of these DEPs in MG tissues were significantly and positively correlated with SCC and DSCC in the milk samples. Among them, CORO1A showed the highest correlation coefficient with SCC and DSCC (Figure 5D). These findings confirm that CORO1A may directly or indirectly regulate immune homeostasis through ion transport and phagosome formation within immune cells, thus contributing to the development of CM in cows.

### 3.5. Distribution and Expression Pattern of CORO1A in MGs of Holstein Cows

**Figure 6:**
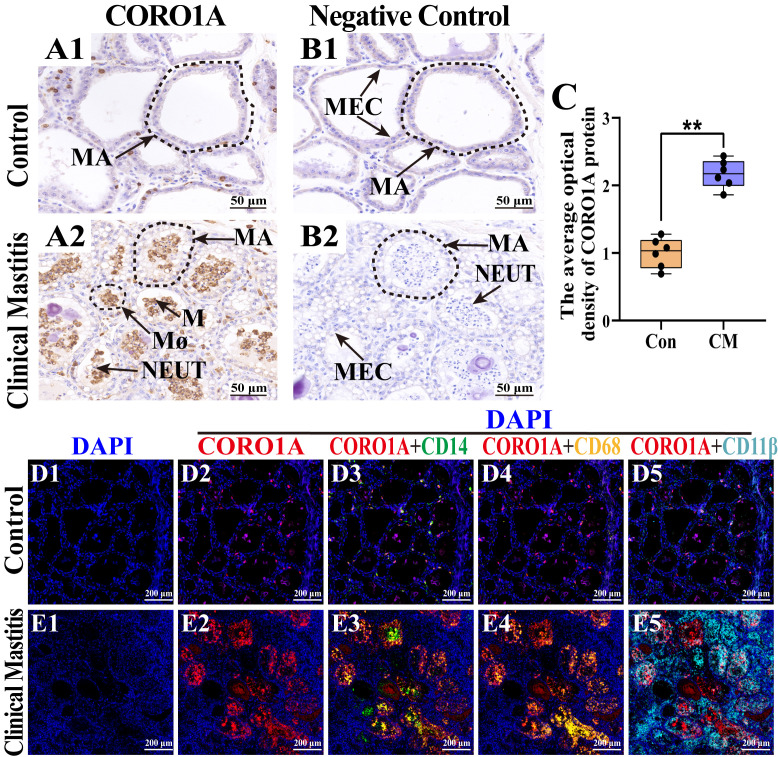
Distribution and expression patterns of CORO1A in MG tissues of the C and CM groups (*n* = 3/group). (**A1**,**A2**) Intracellular location analysis of CORO1A proteins. (**B1**,**B2**) Negative control of the two groups. (**C**) Gray values of positive expression of CORO1A protein quantified using ImageJ software. (**D1**–**E5**) Colocalization analysis of CORO1A and inflammatory cell markers. (**D1**,**E1**) DAPI-stained nuclei of cells. (**D2**,**E2**) CORO1A distribution in the MG tissues. (**D3**–**D5**,**E3**–**E5**) Colocalization analysis of CORO1A and CD14, CD68, and CD11β proteins in MG tissues. MA, mammary alveoli; MECs, mammary epithelial cells; NEUT, neutrophil; M, monocyte; MΦ, macrophage. Scale bars: 50 and 200 μm; 400× and 200×, respectively. All images in this figure are representative. ** represents *p* < 0.01.

The subcellular localization and expression of CORO1A in MG tissue was examined to further investigate their role in immune homeostasis regulation during CM (Figure 6). IHC staining revealed weak CORO1A expression in the MGs of the Con group (Figure 6(A1)). Conversely, CORO1A was widely and strongly expressed in the cytoplasm and nucleus of inflammatory cells in the MGs of the CM group (Figure 6(A2)). No CORO1A staining was detected in the negative controls (Figure 6(B1,B2)). The IOD analysis confirmed significantly higher CORO1A expression in the CM group than in the Con group (*p* < 0.01, Figure 6C). Given the exclusive expression of CORO1A in immune cells within MG tissues, a colocalization analysis with CD14, CD68, and CD11β (markers for monocytes, macrophages, and neutrophils, respectively) was performed. DAPI staining highlighted the nuclei of different cell types in both groups (Figure 6(D1,E1)). In the Con group, the expression of CORO1A was low, with minimal colocalization between CORO1A and the immune cell markers, indicating limited immune cell infiltration in healthy MG tissues (Figure 6(D2–D5)). In contrast, the CM group exhibited elevated CORO1A expression, with significant colocalization with CD14, CD68, and CD11β, confirming its association with immune cell infiltration (Figure 6(E2–E5)). These findings indicate that CORO1A upregulation was closely related to monocyte, macrophage, and neutrophil infiltration, thereby contributing to CM progression and immune homeostasis disorder in dairy cows.

### 3.6. Molecular Mechanism Prediction and Expression Patterns of CORO1A mRNA and Proteins in MGs

**Figure 7:**
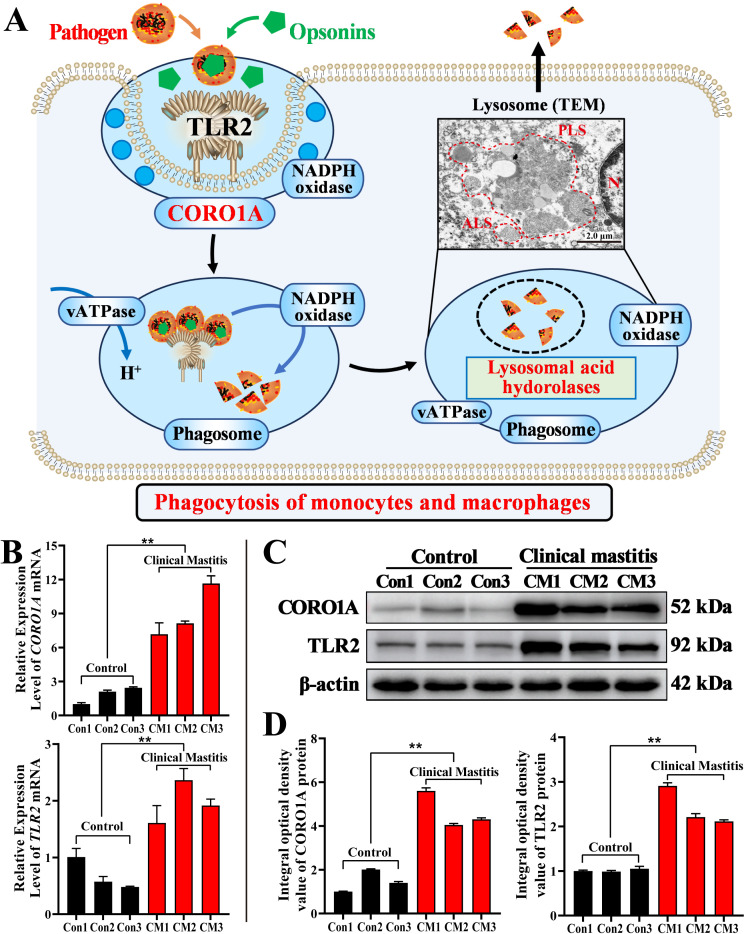
Predicted molecular mechanism and expression patterns of *CORO1A* mRNA and protein in MG tissues of the C and CM groups (*n* = 3/group). (**A**) Potential molecular mechanism of CORO1A-mediated lysosomal phagocytosis in MECs of dairy cows. (**B**) *CORO1A* and *TLR2* mRNA expression levels in MGs of the two groups. *β-actin* mRNA was employed as an internal reference. (**C**) Protein bands of CORO1A and TLR2 monitored using WB; complete WBs are shown in Supplementary Figures S3–S5. (**D**) Relative IOD of CORO1A and TLR2 in MGs. ** represents *p* < 0.01.

Based on the results of this study and the KEGG pathway analysis, we proposed a potential regulatory mechanism (Figure 7A). Specifically, TLR2 recognized pathogens and interacted with opsonin to enhance immune cell phagocytosis [24]. Upon activation, TLR2 recruited CORO1A to the phagosome formation site, indicating that its involvement in the modulation of phagocytosis [31]. Following pathogen engulfment, vATPase facilitated the acidification of the phagosome, promoting its maturation [32,33]. The phagolysosome formation triggered the release of lysosomal acid hydrolases, which contributed to pathogen degradation [34]. Additionally, phagosome acidification, driven by protonation, is essential for killing pathogens. CORO1A may influence this process by modulating phagosome maturation, indirectly enhancing its immune-cell-killing capacity. Finally, the *CORO1A* and *TLR2* mRNAs’ and proteins’ expression in the MGs of both groups were evaluated. The mRNA expression levels of *CORO1A* and *TLR2* in the CM group were significantly upregulated compared to those in the Con group (*p* < 0.01, Figure 7B). TLR2 and CORO1A proteins were detected in each sample from the two groups, showing varying expression levels (Figure 7C). The IOD analysis showed that the TLR2 and CORO1A proteins were significantly upregulated in the CM group compared to those in the Con group (*p* < 0.01, Figure 7D). These findings indicated that CORO1A played a key role in immune homeostasis by promoting phagocytosis during bovine mastitis.

## 4. Discussion

Immune homeostasis refers to the ability of the host to detect and eliminate invading pathogens while maintaining the stability of self-tissues and cells, thereby preventing excessive immune responses or autoimmune damage [35]. In MGs, immune homeostatic regulation is vital for lactation, and mammary gland health directly affects the productivity and milk quality of dairy cows [3,4]. Mastitis is a multifactorial and complex disease influenced by pathogen invasion, immune regulation, tissue repair capacity, and metabolic status. For instance, *Staphylococcus aureus* can disrupt immune homeostasis in mammary tissue through various immune evasion strategies, resulting in chronic inflammation, recurrent infection, and impaired milk secretion [5,17]. This disturbance triggers an imbalanced local immune response characterized by an excessive production of cytokines such as IL-1β, IL-6, and TNF-α, which further amplifies inflammation and contributes to immune dysregulation [26]. Moreover, the negative feedback mechanisms involved in immune homeostasis considerably influence immune cell function and ion balance across different organisms [14]. The disruption of these regulatory mechanisms markedly increases the progression and severity of CM [36]. Therefore, exploring the biomolecular mechanisms underlying immune homeostatic regulation during CM is essential.

Phenotypic observations revealed inflammatory cell infiltration in the mammary tissues and death of MECs during CM. The excessive infiltration of inflammatory cells intensifies the inflammatory response, resulting in the overproduction of ROS and proteases [37]. These factors disrupt mammary tissue architecture, impair lactation, exacerbate pathological damage to the MGs, and disrupt mammary homeostasis [11,38]. The increased activity of autophagic and phagocytic lysosomes indicates the recruitment of mononuclear phagocytes in response to inflammation and infection. Furthermore, the phagolysosomes in macrophages and neutrophils activate phagocytosis to degrade pathogenic microbes and clear infections [39]. The colocalization of autophagosome marker protein LC3 and lysosome marker protein LAMP-2 provides definitive evidence for their fusion, indicating the formation of autophagic lysosomes [40]. Autophagic lysosomes contribute to intracellular homeostasis by degrading damaged organelles and proteins, eliminating inflammation-associated substrates, and modulating antigen presentation and immune cell activation through the activation of autophagy [41].

To elucidate the role of homeostatic regulation in the MG tissues of cows, a DIA proteomic analysis was conducted to identify DEPs. The GO analysis revealed eight immune homeostasis-related BPs and 68 DEPs. These BPs, including leukocyte homeostasis and ion homeostasis, along with the key DEPs, exhibit significant associations with inflammation and immunity. Aberrant activation or suppression of these key proteins can disrupt immune balance. For example, signal transducer and activator of transcription 3 and receptor for activated C kinase 1 dysregulation may lead to chronic inflammation or immunosuppression, thereby disrupting immune homeostasis in mammary tissues [19]. Caveolin 1 contributes to ion homeostasis by regulating calcium influx and signal transduction [42] and plays a role in mastitis by modulating leukocyte homeostasis and inflammatory responses [43]. Heme oxygenase 1 supports iron homeostasis in MG tissues by regulating iron ion release and metabolism, thereby preventing ferroptosis-induced mammary inflammation [23]. Notably, CORO1A is involved in most of the BPs related to immune homeostasis, which highlights its critical involvement in the development of cow mastitis. Pathway analysis revealed that CORO1A directly modulated immune cell and ion homeostasis through phagosome formation and the tuberculosis pathway. This enrichment reflects the overlap of immune-related signaling components such as TLRs and NF-κB, which are activated in both mastitis and tuberculosis, rather than disease specificity. CORO1A facilitates immune cell migration to the inflamed sites by regulating cytoskeletal reorganization. This action aids in clearing inflammatory foci [44]. Taken together, CORO1A may regulate bovine mammary homeostasis in cows by facilitating ion transport and phagosome formation in immune cells.

Although CORO1A has not been widely studied in mastitis, and baseline expression data in healthy mammary glands are currently lacking, previous studies have linked it to immune signaling and phagocytosis. Studies have shown that the loss of CORO1A in macrophages inhibits agonist-induced actin polymerization, thereby impairing macrophage migration and phagocytosis [45]. Furthermore, CORO1A promotes neutrophil proliferation and adhesion, which are essential for the phagocytosis and clearance of pathogens in innate immunity [46]. In this study, CORO1A was expressed in immune cells within MG tissues and was localized in the monocytes, macrophages, and neutrophils. These findings suggest that CORO1A-mediated phagocytic activity may contribute to the regulation of mammary immune homeostasis. Although not directly examined in this study, CORO1A may influence phagosome acidification—a protonation-dependent process essential for pathogen killing—by promoting phagosome maturation. Moreover, a potential interaction between CORO1A and the TLR2 signaling pathway may enhance the bactericidal activity of macrophages during phagocytosis, thereby facilitating pathogen clearance at infection sites [44,47]. TLR2 regulates the activity of multiple immune cell subsets, such as macrophages and dendritic cells, thereby playing an essential role in controlling inflammation and maintaining immune homeostasis [48]. These observations indicate that CORO1A and TLR2 might exhibit synergistic effects in regulating immune homeostasis during mastitis. However, our results showed that the mRNA and protein expression of *CORO1A* and *TLR2* was abnormally upregulated during mastitis. These findings suggested that differences in the activation levels of CORO1A and TLR2 might be associated with dual effects. Specifically, CORO1A/TLR2 effectively recruited immune cells to mediate pathogen clearance upon the microbial invasion of MG tissues; conversely, the marked upregulation of both CORO1A and TLR2 under immune hyperactivation conditions was associated with disrupted immune homeostasis, aggravated inflammatory cascades, and significant parenchymal damage. Further investigation into the CORO1A signaling axis and its downstream effectors may provide novel strategies for restoring MG immune homeostasis and treating CM.

Some limitations of this study should be acknowledged. First, all CM cases analyzed were caused by *Staphylococcus aureus*, potentially limiting the generalizability of our findings to mastitis induced by other pathogens. Second, further mechanistic studies employing both in vitro and in vivo models are required to delineate the dynamic role of CORO1A in regulating immune homeostasis and inflammation. Nonetheless, the aberrant activation of the CORO1A observed in mammary gland tissues in this study provides meaningful insights into the immunoregulatory processes underlying clinical mastitis.

## 5. Conclusions

Significant inflammatory cell infiltration and activation of autophagic and phagocytic lysosomes were observed in the MG tissue during mastitis, indicating the disruption of immune homeostasis. The bioinformatics analysis identified CORO1A among eight leukocyte and ion homeostasis-related BPs and 68 DEPs, with a pathway analysis suggesting its role in activating the immune and phagocytic responses. IHC and IF staining localized CORO1A in the monocytes, macrophages, and neutrophils, while a mechanistic analysis revealed that TLR2 recruits CORO1A to the phagosome, thereby enhancing phagocytic activity. Notably, *TLR2* and *CORO1A* mRNA and protein levels were significantly upregulated compared to those in the Con group. These findings indicate that CORO1A regulates immune homeostasis in CM through phagocytosis, thus providing a foundation for understanding immune regulation in Holstein cows. Future studies should explore the dynamic roles of CORO1A in immune cell migration, phagosome maturation, and inflammation resolution using both in vitro and in vivo models.

## Data Availability

The original contributions presented in this study are included in the article/Supplementary Material. Further inquiries can be directed to the corresponding author(s).

## Supplementary Materials

The following supporting information can be downloaded at: https://www.mdpi.com/article/10.3390/biom15060827/s1, Table S1: The used antibodies and dilution ratio in the present study. Table S2: qRT-PCR primer sequences. Table S3: The biological processes (BPs) associated with homeostasis from DIA proteomics. Table S4: The 2 Pathways and 74 DEPs RAW Data identified from DIA proteomics. Table S5: Analysis of KEGG pathways associated with CORO1A, BAX and BAK1. Table S6: The significant candidate DEPs related to homeostasis based on BPs and pathways analysis. Figure S1: Bacteriological culture of milk samples. Figure S2: Blue Plus II Protein Marker. Figures S3–S5: Whole Western blots of TLR2, CORO1A, and β-actin, respectively.
