# Supplementary figures and images for: Role of CORO1A in Regulating Immune Homeostasis of Mammary Glands and Its Contribution to Clinical Mastitis Development in Dairy Cows
Source: Biomolecules. 2025 Jun 6;15(6):827. doi: 10.3390/biom15060827 (PMC12191025; doi:10.3390/biom15060827)

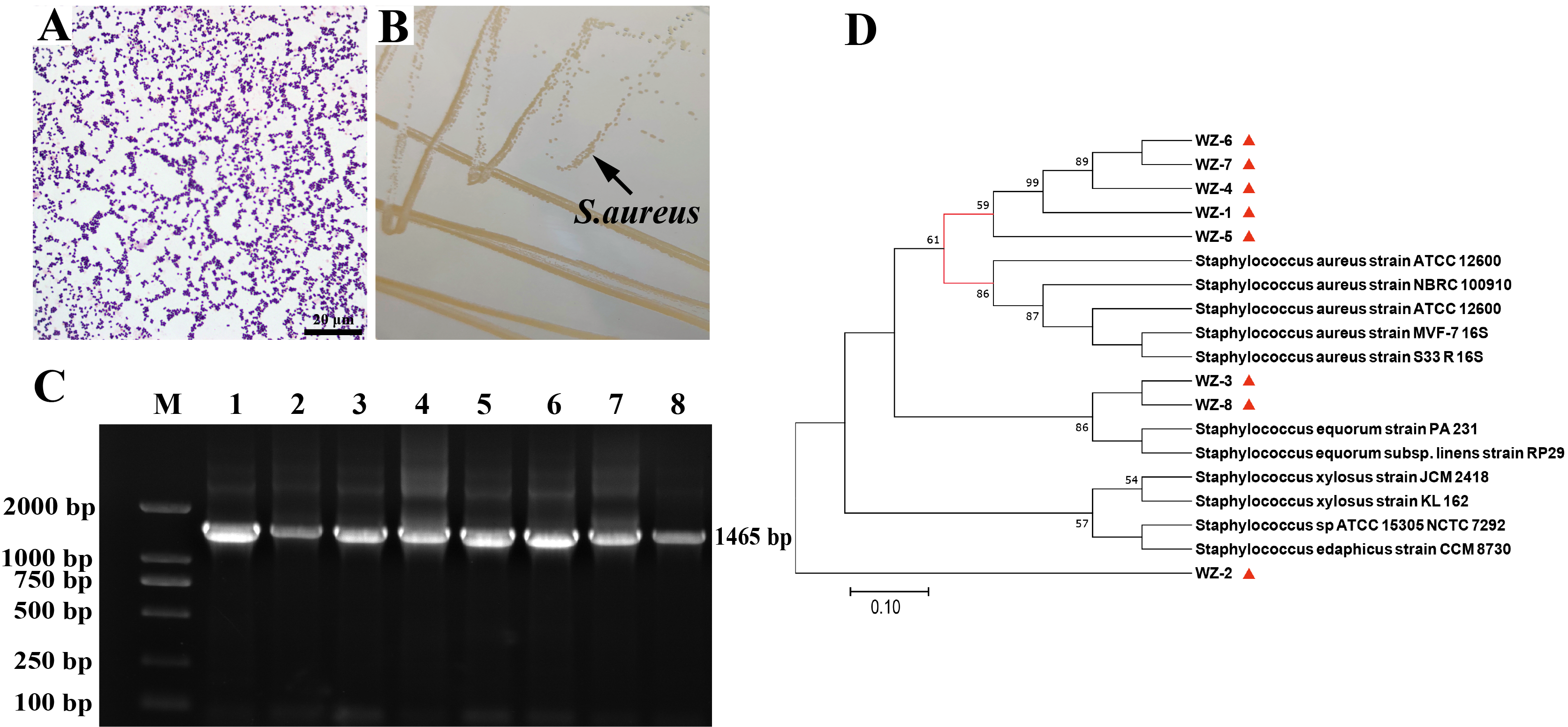

Supplement: Supplementary file 1 [file biomolecules-15-00827-s001.zip › Figure S1 (Bacteriological culture of milk samples).tif]

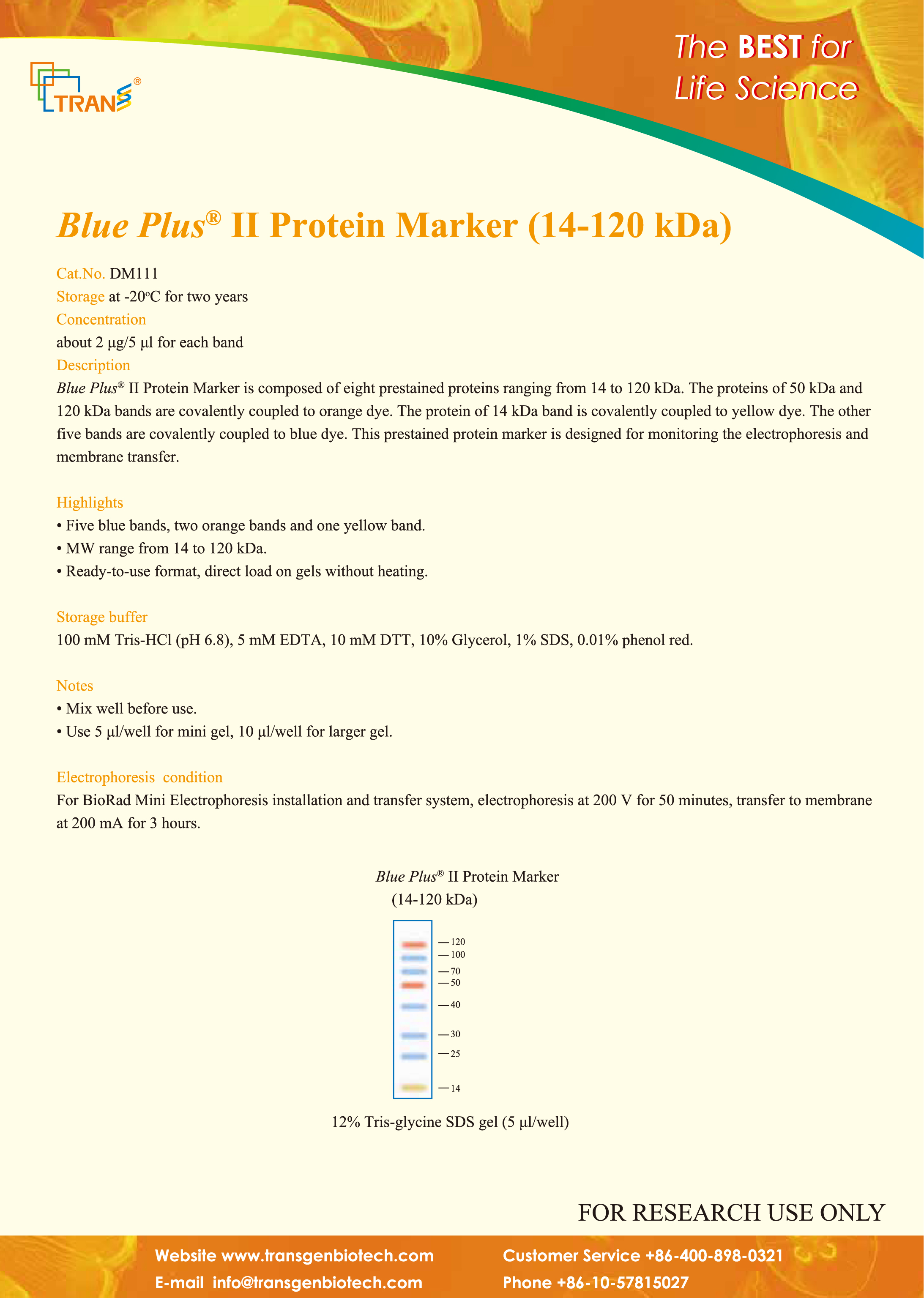

Supplement: Supplementary file 1 [file biomolecules-15-00827-s001.zip › Figure S2 (Blue Plus ll ProteinMarker instructions).jpg]

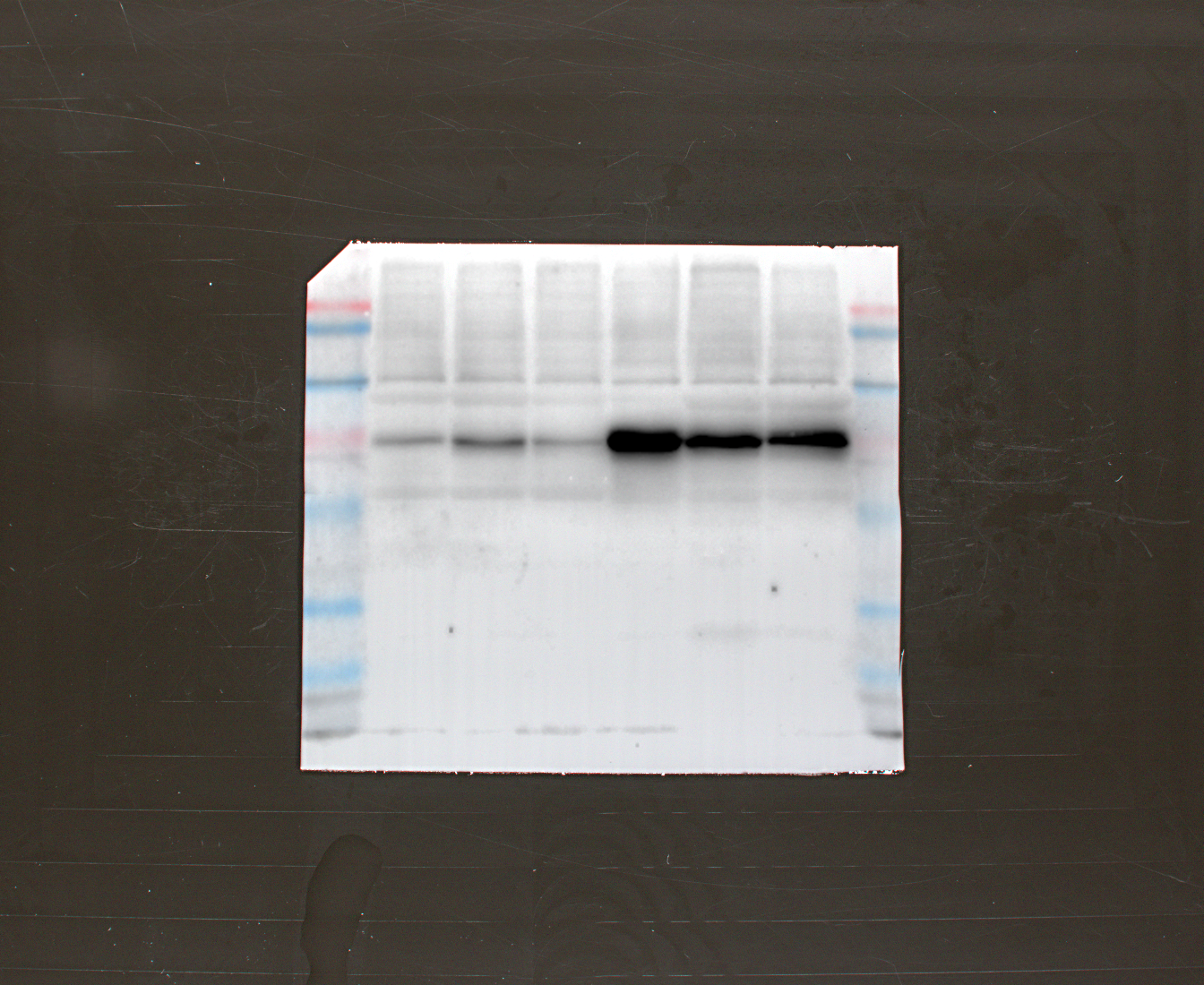

Supplement: Supplementary file 1 [file biomolecules-15-00827-s001.zip › Figure S3 (CORO1A 52kDa).Tif]

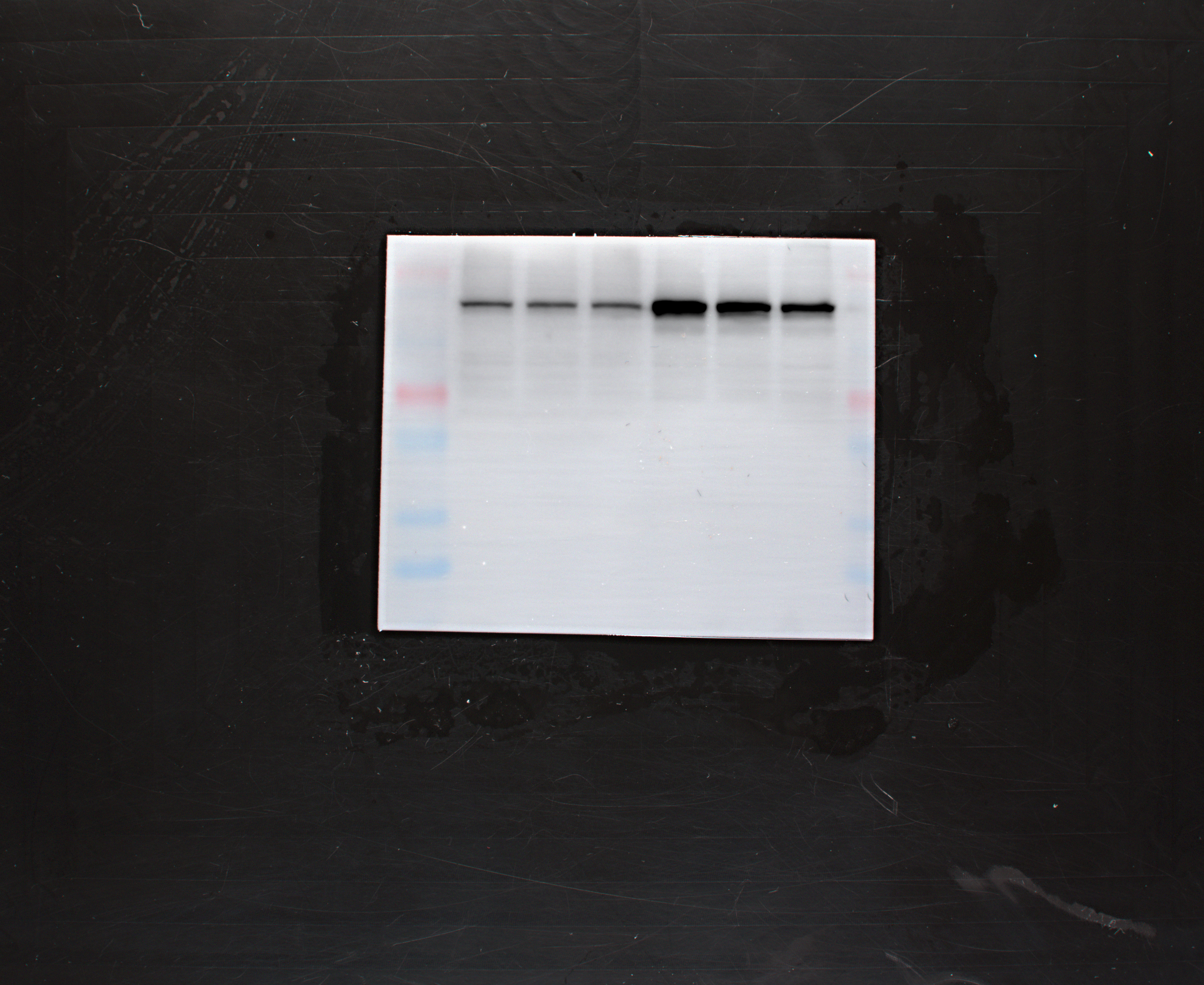

Supplement: Supplementary file 1 [file biomolecules-15-00827-s001.zip › Figure S4 (TLR2 92kDa).Tif]

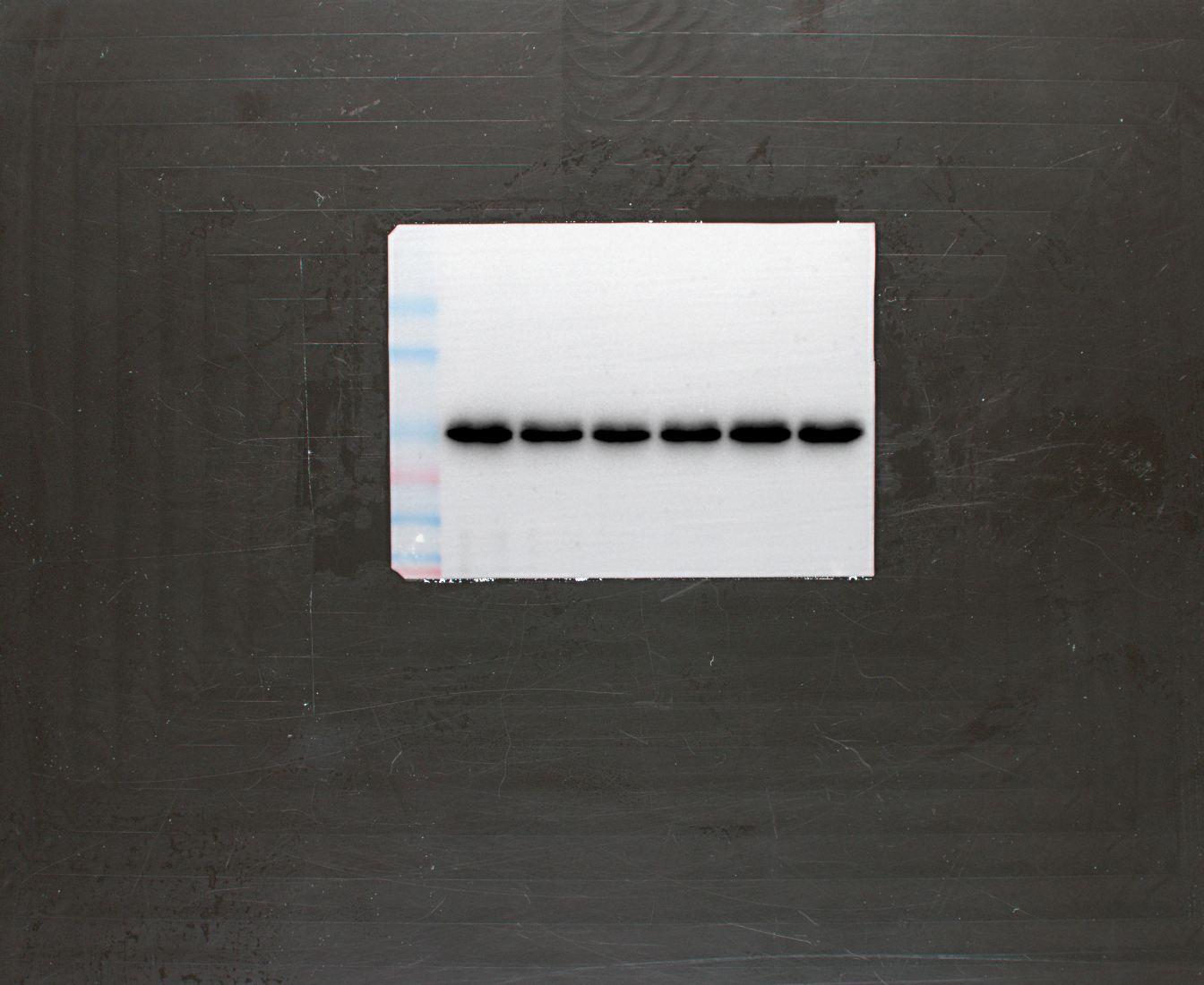

Supplement: Supplementary file 1 [file biomolecules-15-00827-s001.zip › Figure S5 (β-actin 42kDa).Tif]
